# Correlation between the quality of nursing handover, job satisfaction, and group cohesion among psychiatric nurses

**DOI:** 10.1186/s12912-022-00864-8

**Published:** 2022-04-11

**Authors:** Bin Wang, Guiyuan Zou, Mei Zheng, Chen Chen, Weiyu Teng, Qinghua Lu

**Affiliations:** 1 Department of Psychiatric, Shandong Provincial Hospital affiliated to Shandong First Medical University, Jinan, 250000 Shandong China; 2 Department of Psychiatric, Shandong Mental Health Center, Shandong University, 49 Wenhua East Road, Jinan, 250014 Shandong China

**Keywords:** Psychiatric nurses, Quality of nursing handover, Job satisfaction, Group cohesion

## Abstract

**Background:**

Nursing handovers are a critical component of patient safety. Researchers have performed many primary studies in this field, mainly reporting findings from changes in nursing handover patterns. However, few quantitative studies have explored the factors that influence handover quality. Therefore, this study aimed to investigate the quality of handovers and explore the associations between handover quality, job satisfaction, and group cohesion among psychiatric nurses.

**Methods:**

This cross-sectional study included 186 registered psychiatric nurses from a Chinese hospital, who responded to the Handover Evaluation Scale, McCloskey/Mueller Satisfaction Scale, and Group Cohesion Scale. Bootstrap analyses were used to evaluate the mediating effect between variables.

**Results:**

The average item score for handover quality was (5.85 ± 1.14), and job satisfaction and group cohesion could predict the variance of handover quality. Job satisfaction could partially mediate between group cohesion and handover quality, and the value of the mediating effect was 45.77%.

**Conclusion:**

The quality of psychiatric nursing handovers has enhanced space. Thus, hospital managers should take various measures to strengthen group cohesion and promote job satisfaction, both of which help improve the quality of psychiatric nursing handovers.

## Background

Patient safety is a serious global public health issue, as adverse events are common in health facilities, especially psychiatry [1–3]. Psychiatric nurses care for psychiatric patients with mood disturbances and psychotic symptoms that may experience sudden, unexpected, and illogical agitation. Adverse psychiatric events, including violence, suicide, escape, falls, choking, and medication errors, have been widely reported [4, 5]. Several studies have shown that patient safety failures result from human factors concerning communication, teamwork, and mental health among healthcare professionals [ 4, 6, 7]. Promoting high-quality continuous care that can protect patients from harmful risks related to healthcare practices is a core component of patient safety. Among others, nursing handovers as a critical segment of patient safety deserve extensive attention [7, 8]. A nursing handover is a real-time process in which patient-specific information is passed between nurses to ensure the continuity and safety of patient care [9]. Nursing handovers are practiced daily in many ways in all institutional healthcare settings. Nursing handovers play a central role in achieving well-coordinated care, ensuring work continuity, delivering relevant clinical information to the follow-up care team, and sharing patient-related messages in an accurate and timely manner [10, 11]. Furthermore, an inadequate handover implies adverse events, delays, inappropriate treatment, or lack of care. To date, many studies have reported the benefits of nursing handover quality improvement projects aimed at enhancing patient safety in clinical practice [8, 10]. A systematic review suggested the effectiveness of using Situation, Background, Assessment, and Recommendation intervention as a handover tool and communication on patient safety [8]. Researchers have performed many primary studies in the field, mainly reporting findings from changes in nursing handover patterns. However, a previous survey found that interruptions are potentially a significant barrier to the delivery of effective handovers, and the mean interruption time was 0–4 min during a 15 min handover [12]. Liu et al. reported that the handover evaluation of nurses in general hospitals was 6.35 ± 0.74, indicating a higher level of quality of handover [13]. However, few quantitative studies have investigated the handover evaluation of psychiatric nurses and the analysis of the influencing factors.

Team is an important department of the healthcare system, which performs an active function in healthcare practices. Group cohesion is a phenomenon that determines how well a team holds together [14]. It has been defined as a subjective perception of bonding, working together toward common goals, mutual acceptance, and group affiliation [15]. Group cohesion is integral to cultivating a supportive work atmosphere and high-quality person-centered cultures of care in the modern healthcare setting [16, 17]. Studies have found that group cohesion was contributive to nurses in terms of occupational performance and job satisfaction [17–19]. However, few studies revealed the association between group cohesion and quality of nursing handover among psychiatric nurses.

Job satisfaction is a multidimensional construct related to work engagement [20, 21]. Job satisfaction is also a predictor of psychological distress and other organization outcomes [22]. High job satisfaction is vital for nurses to ensure the high-efficiency delivery of service quality. Job satisfaction can strongly predict positive job performance such as being active at work, pursuing new goals, and developing good relationships. Conversely, workers with lower job satisfaction display high nurse turnover rates and poor quality patient care [23, 24].

Summarizing the findings of previous research, group cohesion was not only related to good psychosomatic experience but also a key indicator of professional performance for nurses. Nursing handovers are a form of professional performance that undoubtedly correlate with job satisfaction. Based on these facts, we hypothesized that job satisfaction could mediate the association between group cohesion and quality of psychiatric nursing handovers.

The purpose of this cross-sectional study was to achieve the following: (1) to investigate the status of psychiatric nursing handovers; (2) to examine the relationships between group cohesion, job satisfaction, and quality of psychiatric nursing handovers; and (3) to explore the mediating effect of job satisfaction on the relationship between group cohesion and psychiatric nursing handovers.

## Methods

### Design, sample, and participants

This correlational quantitative study was conducted in a tertiary psychiatric hospital in Shandong Province, China. Convenience sampling was used to survey registered nurses. We calculated the sample size using the following formula: N = (μα S/δ)^2^ (where *μ*_*α*_ = 1.96, and the S/δ value = 6 according to the pilot study), and considering 20% nonresponse rates, we found that the minimum sample size was 166. The inclusion criteria for the participants were as follows: (a) with a practice license for nurses of the People’s Republic of China; (b) directly involved in a nursing unit, including a psychiatric or psychological department. The study was approved by the Ethics Committee of Shandong Mental Health Center (2018R23). The procedures were conducted per the ethical standards of the 1964 Declaration of Helsinki.

### Data collection procedures

Data were collected in December 2019 using online questionnaires via Survey Star (https://www.wjx.cn). Recruitment documents and links to online questionnaires were distributed through social network services, through which head nurses communicated. All participants were informed of the study through recruitment documents. Participants could choose to participate by clicking on the link to the online survey. To avoid replication, the IP address of every respondent was checked when online questionnaires were received. All questionnaires required 10–15 min to complete. Finally, the questionnaire was distributed to 206 invited nurses, and 186 questionnaires were successfully completed, giving a response rate of 90.29%.

### Measures

#### Participants’ sociodemographic characteristics

Participants’ sociodemographic information including age, sex, level of education, marital status, professional title, years of work, and job type was collected.

#### Quality of nursing handovers

The 13-item self-rating Handover Evaluation Scale originating from O′Connell’s study was used to assess the level of quality of nursing handovers, which measures three dimensions: quality of information (six items), interaction and support (four items), and efficiency (three items) [24]. Each item was rated on a Likert-type scale, where 1 means do not agree at all, and 7 means agree completely. Scores were summed to obtain a total score ranging from 13 to 71, with higher scores indicating higher levels of quality nursing handovers [24]. The Cronbach’s alpha value for the Chinese version translated by Liu et al. was 0.91 and the content validity was 0.92 [25]. Content and face validities were confirmed using a qualitative method by asking for the opinions of five psychiatric nursing experts, and the content validity was 0.93. Cronbach’s α was 0.96 for the overall HES in our research.

#### Job satisfaction

The McCloskey/Mueller Satisfaction Scale developed by McCloskey was used to measure the degree of job satisfaction [20, 26]. This scale contains 31 items, and each item was rated on a 5-point Likert-type scale ranging from 1 (strongly disagree) to 5 (strongly agree). The total score ranges from 31 to 155, with a higher total score indicating a higher level of job satisfaction. An item with an average score > 3.03 indicates low job satisfaction. The Chinese version of the McCloskey/Mueller Satisfaction scale has been widely used and has demonstrated satisfactory reliability and validity for the Chinese sample [27, 28], and the Cronbach’s α in this study was 0.97.

#### Group cohesion

Group cohesion was measured using the 12-item Group Identification Scale developed by Henry [14]. Each item was rated on a 5-point scale ranging from 0 (strongly disagree) to 7 (strongly agree). The total score for summing up the 12-item score and the higher score forecasted higher group cohesion. The Chinese version of the Group Identification Scale has proven valid and reliable (Cronbach’s α = 0.88) for the Chinese sample [29, 30]. Five psychiatric nursing experts confirmed the face and content validity of this scale. The 12-item Group Identification Scale showed an acceptable level of reliability (Cronbach’s α = 0.92).

### Statistical analysis

Statistical Package for the Social Sciences (SPSS) software version 22 was used for data analysis. Descriptive statistics for the quality of nursing handover, group cohesion, job satisfaction, and sociodemographic information were collected. A *t*-test or analysis of variance was used to assess the differences in the quality of nursing handovers. Pearson’s correlation was used to examine the relationship between the quality of nursing handovers, group cohesion, and job satisfaction. Hierarchical regression analysis was conducted to explore the mediation of job satisfaction between group cohesion and quality of nursing handover per Baron and Kenny’s method [31] and standardized estimate (β), F, R^2^ were provided. Finally, the SPSS Macro PROCESS plugin was proposed by Preacher and Hayes to examine the statistical significance of mediation [32]. For all analyses, a *p*-value < 0.05 was considered statistically significant.

## Results

### Sociodemographic characteristics of the participants and distribution of quality of nursing handover in categorical items

As shown in Table 1, the sample comprised 144 (77.4%) women and 42 (22.6%) men. The mean age of the participants was (36.72 ± 6.63) years ranging from 25 to 59 years. Regarding employment duration, 6 (3.2%) have worked as nurses for 1–3 years, 15 (8.1%) for 3–5 years, 51 (27.4%) for 5–10 years, and 114 (61.3%) for > 10 years. In terms of education, 180 (96.8%) of the nurses had earned a bachelor’s degree or higher; 163 were married and 155 (83.3%) were permanent nurses. Regarding professional titles, 13 (7.0%) had a senior title, 92 (49.5%) had an intermediate title, and 81 (43.5%) had a junior title. There were no significant differences between the quality of nursing handovers and sociodemographic characteristics.

**Table 1:** Sociodemographic characteristics of the participants and distribution of quality of nursing handover in categorical items (*n* = 186)

Variables	n (%)	Quality of nursing handover	t/F value
Sex			0.202
Male	42(22.6)	75.74 ± 16.65	
Female	144(77.4)	76.21 ± 14.24	
Age			0.394
≤30 years	37(19.9)	77.92 ± 17.5	
31 ~ 45 years	126(67.7)	75.49 ± 14.48	
> 45 years	23(12.4)	76.52 ± 11.96	
Marital status			0.002
Single	23(12.4)	75.96 ± 15.31	
Married	163(87.6)	76.12 ± 14.74	
Education level			0.017
Bachelor’s degree or above	180(96.8)	75.93 ± 14.81	
Junior school or under	6(3.2)	81.33 ± 13.53	
Employment duration			1.515
1 ~ 3 years	6(3.2)	81.00 ± 6.72	
3 ~ 5 years	15(8.1)	81.60 ± 9.93	
5 ~ 10 years	51(27.4)	73.37 ± 17.99	
> 10 years	114(61.3)	76.34 ± 13.83	
Professional title			1.614
Junior title	81(43.5)	74.09 ± 17.42	
Intermediate title	92(49.5)	77.26 ± 12.69	
Senior title	13(7.0)	80.46 ± 7.90	
Job type			
Temporary nurse	31(16.7)	79.61 ± 10.64	0.510
Permanent nurse	155(83.3)	75.40 ± 15.40	

### Scores and correlations among group cohesion, job satisfaction, and quality of nursing handover

Descriptive results for the quality of nursing handovers, group cohesion, and job satisfaction are depicted in Table 2. The average item score for quality of nursing handover was (5.85 ± 1.14). Among the three subscales of quality of nursing handover, the average item score ranged from high to low in the following order: quality of information (6.15 ± 1.17), interaction and support (5.73 ± 1.31), efficiency (5.43 ± 1.25). The mean item scores for group cohesion and job satisfaction were (5.75 ± 0.94) and (2.88 ± 0.72), respectively, indicating moderate group cohesion and low job satisfaction.

**Table 2:** Mean, Standard Deviations (SD) and Correlation of Variables(r)

Variables	Mean ± SD(^−^χ ± s)	Quality of nursing handover	Quality of information	Interaction and support	Efficiency	Group cohesion	Job satisfaction
Quality of nursing handover	5.85 ± 1.14	1	–	–	–	–	–
Quality of information	6.15 ± 1.17	0.946*	1	–	–	–	–
Interaction and support	5.73 ± 1.31	0.928*	0.802*	1	–	–	–
Efficiency	5.43 ± 1.25	0.871*	0.734*	0.758*	1	–	–
Group cohesion	5.75 ± 0.94	0.393*	0.348*	0.364*	0.387*	1	–
Job satisfaction	2.88 ± 0.72	0.544*	0.465*	0.505*	0.567*	0.390*	1

Notes:* *P*<0.01

Table 2 shows the correlations between the study variables. Group cohesion was positively correlated with the quality of nursing handover (r = 0.393, *p* < 0.01) and job satisfaction (r = 0.393, *p* < 0.01), and job satisfaction was positively correlated with the quality of nursing handover (r = 0.544, *p* < 0.01).

### Results of hierarchical linear regression analysis

As shown in Table 3, group cohesion was significantly associated with the quality of nursing handovers (β = 0.393, *P* < 0.001), explaining 15.4% of the variance in nursing quality. In Step 2, job satisfaction was a positive predictor of the quality of nursing handover (β = 0.213, *P* < 0.001), accounting for an additional 18% of the variance in the quality of nursing handover. When job satisfaction was added to the regression model, the relationship between group cohesion and quality of nursing handovers was significantly weakened. Finally, group cohesion and job satisfaction were correlated with the quality of nursing handovers, explaining 33.4% of the variance in the quality of nursing handovers. A collinearity diagnosis for the final hierarchical model, using the tolerance and variance inflation factor (VIF), demonstrated that the variables were not overly correlated.

**Table 3:** Hierarchical Linear Regression Analysis Results

Variables	Quality of nursing handover		Collinearity Statistics	
	Step1(β)	Step2(β)	Tolerance	VIF
Group cohesion	0.393*	0.213*	0.848	1.134
Job satisfaction		0.461*	0.848	1.179
F	33.540*	45.982*		
R^2^	0.154	0.334		

Notes:* *P*<0.01

After adding job satisfaction in Step 2, the regression coefficients for group cohesion (from β = 0.393 to β = 0.213, *p* < 0.01) decreased. Following Baron and Kenny’s procedures for mediational hypotheses [31], we can speculate that job satisfaction partially mediates the relationship between group cohesion and the quality of nursing handovers. The results of the bootstrapping method (Table 4) showed that the path coefficient of the indirect effect of group cohesion on job satisfaction was 0.2178 (95%CI:0.1236, 0.3484). The indirect effect of group cohesion on the quality of nursing through job satisfaction accounted for 45.8% of the total effect.

**Table 4:** Results for the direct and indirect effects of group cohesion on quality of nursing handover with job satisfaction as mediator

Mediation model hypothesis	Effects	Point Estimate	95% CI		*P* value
			LL	UL	
Group cohesion-	Total effect	0.4760	0.3138	0.6381	<0.001
Job satisfaction-	Direct effect	0.2581	0.1015	0.4148	<0.001
Quality of nursing handover	Indirect effect	0.2178	0.1236	0.3484	<0.001

## Discussion

To the best of our knowledge, this is the first study to investigate the status of quality of handovers and explore the factors influencing the quality of handovers among Chinese psychiatric nurses. This is also the first study to examine job satisfaction’s mediation between group cohesion and the quality of psychiatric nursing handovers. The main findings were as follows: the score of quality of nursing handover was above the medium level, quality of nursing handover was associated with group cohesion and job satisfaction, and mediation analysis showed that job satisfaction was a partial mediator between group cohesion and the quality of nursing handover.

The quality of nursing handovers has become an international priority in both research and clinical fields for patient safety [33–35]. The current research show that the score of quality of psychiatric nursing handover is (5.85 ± 1.14), indicating that the quality of nursing handover is good. However, the score of quality of nursing handovers is significantly lower than that of general hospital nurses [13], indicating that there is room for improvement for psychiatric nurses. Our study did not find a significant association between participants’ sociodemographic characteristics and handover quality score, which disagrees with previous findings. Liu revealed that the scores of nurses with a bachelor’s degree or greater were significantly higher than those of nurses with junior school or lower [13]. This may have occurred because the percentage of nurses with a bachelor’s degree or higher was 96.8%. The average item score of each dimension from high to low was as follows: quality of information, interaction and support, and efficiency. Adverse events, such as violence, suicide, escape, choking, and falls, occur frequently in psychiatric hospitals. Therefore, timely identification and delivery of risk assessment information are extremely important for the quality of information. The dimension of efficiency score was the lowest, suggesting that psychiatric nurses should control the content and timing of handovers. Prior research has revealed that delivering excessive information irrelevant to patient care can reduce work efficiency [36]. Relevant studies have also found that nurses cannot effectively cope with working confusion, resulting in low organizational support and high burnout, thereby affecting the level of the interaction and support dimension [13].

Group cohesion is a decisive factor affecting individual and organizational performance, and it is frequently emphasized in the field of social and organizational psychology [19, 30]. The score of group cohesion in this study was (5.75 ± 0.94), indicating good group identification. A possible explanation is that psychiatric nurses were prone to developing a team spirit in dealing with sudden, unexpected, and illogical agitation behaviors, which might cause more group cohesion. A significant positive correlation between group cohesion and the quality of nursing handovers was established in the present study. Relevant studies have revealed that individuals with high group cohesion usually display more cooperation, vigor, concentration, communication, and less burnout in daily work, whereas higher group cohesion is conducive to promoting nurses’ clinical communication skills and professional knowledge, thereby resulting in higher levels of quality of nursing handover [37]. These findings highlight the need to develop strategies for improving group cohesion among psychiatric nurses.

The total score for job satisfaction in the present study was slightly higher than Chen’s finding [38]. This may have occurred because the practice environment of psychiatric nurses has significantly improved due to the recent concern of the government and society. However, we also found that the global score of job satisfaction among psychiatric nurses was poor, that is, lower than the lowest index of job satisfaction. Related research has found that job satisfaction among Chinese nurses differs due to differences in welfare, promotion, social prejudice, working years, and region [22, 39]. Consistent with previous research showing that job satisfaction is not only an outcome of occupational stress but also benefits psychosomatic health and other organizational outcomes (e.g., absenteeism and performance) [22–24], the results of this survey confirmed that job satisfaction was also the most important predictor of quality of nursing handovers. Job satisfaction explained 18.0% of the variance in nursing handover quality. Furthermore, prior surveys have confirmed the protective and positive effects of job satisfaction on nursing handover quality [40, 41]. Individuals with high job satisfaction and behaviors, such as being active in their job and maintaining good interpersonal relationships, were discovered. On the contrary, low job satisfaction was associated with the following behaviors: reduced work pace, absenteeism, resignation, and negative emotion, which caused low-quality nursing handovers.

The most unanticipated finding in the current study was the mediating effect of job satisfaction on the association between group cohesion and quality of nursing handovers among psychiatric nurses, which suggests that group cohesion not only directly influences the quality of nursing handovers but also indirectly the quality of nursing handovers via job satisfaction. Prior surveys have revealed an association between group cohesion and job satisfaction; in particular, group cohesion is beneficial to nurses, both in terms of job satisfaction [42]. Other studies have shown that nurses participate in power-sharing activities, foster a collaborative work environment, and demonstrate greater job satisfaction. High group cohesion may enhance job satisfaction, thereby improving the quality of nursing handovers. The indirect effect of group cohesion on the quality of nursing handovers through job satisfaction accounted for 45.8% of the total effect, indicating that job satisfaction might play a more important role in improving the quality of nursing handovers.

This study has some limitations. First, we could not define causality between group cohesion, job satisfaction, and quality of nursing handovers due to the cross-sectional research design. Second, all data were obtained from self-report questionnaires; thus, reporting bias cannot be avoided. Third, the limited sample size and inclusion of only a single psychiatric institution’s nurses in the survey can be considered as limitations. Further studies that recruit participants from wider areas are needed to verify the results of the current research. Moreover, further studies should be conducted to examine whether other personal and social factors (organizational support, work pressure, self-efficacy, and burnout) affect the quality of psychiatric nursing handovers.

## Conclusions

The quality of psychiatric nursing handovers has enhanced the space. The findings of this study indicate that group cohesion and job satisfaction are positively related to the quality of psychiatric nursing handovers. The current study also showed job satisfaction mediation between group cohesion and the quality of psychiatric nursing handovers. These findings call for health managers to take various measures to strengthen group cohesion and promote job satisfaction, both of which are helpful for the quality of psychiatric nursing handovers. Specifically, health managers should commit to cultivating a comfortable supportive working environment and decreasing workloads, promoting a balance between demands and rewards, and increasing their income and job autonomy.

## Data Availability

The datasets generated and analyzed for the current study are not publicly available due to IRB agreements but are available from the corresponding author on reasonable request.
